# Man’s Worst Friend?: TCDD and Male Reproductive Effects

**Published:** 2006-11

**Authors:** Julia R. Barrett

Animal studies indicate that 2,3,7,8-tetrachlorodibenzo-*p*-dioxin (TCDD) and related compounds are reproductive and developmental toxicants, but effects of human exposures are unclear. Five months after the January 1999 accidental contamination of animal feed with polychlorinated biphenyls and TCDD in Belgium, a research team collected blood and semen samples from 101 men aged 20 to 40 living in Antwerp and Peer **[*EHP* 114:1670–1676; Dhooge et al.]**. The resulting analysis is the first to link exposure to TCDD and dioxin-like compounds to male reproductive effects in the general population.

Blood sample hormone analysis yielded measurements for total testosterone, luteinizing hormone, follicle-stimulating hormone, sex hormone-binding globulin, total 17β-estradiol, and inhibin B. Free testosterone was calculated from total testosterone and sex hormone–binding globulin values. Blood samples were also used to assay for compounds with the ability to bind to aryl hydrocarbon receptor, the target of TCDD; these results were expressed as a TCDD equivalent (TEQ). Semen analysis included volume, sperm concentration and morphology, and total sperm counts.

Serum TCDD levels increased significantly with age and consumption of eggs, fish, and chicken. Rising TEQ values were associated with greater sperm concentration and reduced semen volume and total testosterone. These results could indicate that dioxin-like compounds decrease testosterone levels in the blood and consequently interfere with the secretory function of the seminal vesicles or prostate. The researchers saw no significant relationships between TEQ and any of the other hormone levels or with total sperm count or morphology. TEQ was also positively correlated with egg consumption, a possible reflection of the food contamination accident.

Based on their analyses, the researchers conclude that TCDD and other compounds that bind to the aryl hydrocarbon receptor do not affect sperm creation. Whether decreased gland secretion has an impact on the fertilizing capacity of the sperm cell cannot be determined from these results.

## Figures and Tables

**Figure:**
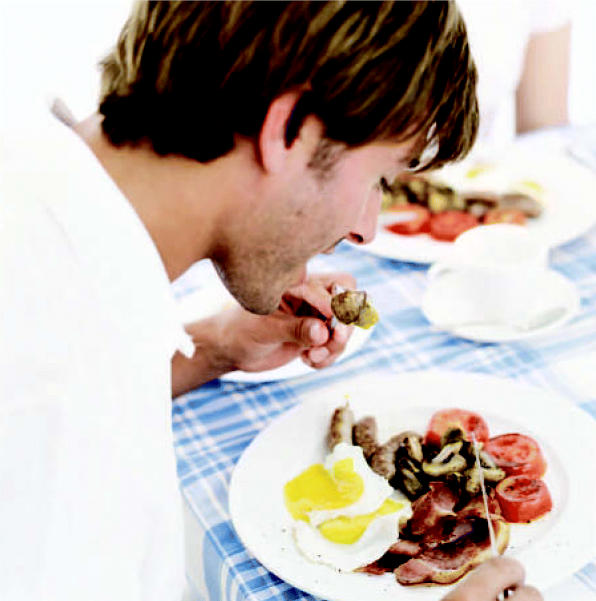
Breakfast of losers? Men who ate eggs, fish, and chicken following a food supply dioxin contamination incident showed decreased semen volume and testosterone.

